# Resin infiltrant with antibacterial activity: effects of incorporation of DMAHDM monomer and NACP on physical and antimicrobial properties

**DOI:** 10.1590/1678-7757-2024-0263

**Published:** 2024-12-02

**Authors:** Ana Ferreira SOUZA, May Anny Alves FRAGA, Américo Bortolazzo CORRER, Flávio Henrique Baggio AGUIAR, Giselle Maria MARCHI

**Affiliations:** 1 Universidade Estadual de Campinas Faculdade de Odontologia de Piracicaba Departamento de Odontologia Restauradora Piracicaba SP Brasil Universidade Estadual de Campinas - UNICAMP, Faculdade de Odontologia de Piracicaba, Departamento de Odontologia Restauradora, Piracicaba, SP, Brasil.; 2 Universidade Estadual de Campinas Faculdade de Odontologia de Piracicaba Divisão de Materiais Dentários Piracicaba SP Brasil Universidade Estadual de Campinas - UNICAMP, Faculdade de Odontologia de Piracicaba, Divisão de Materiais Dentários, Departamento de Odontologia Restauradora, Piracicaba, SP, Brasil.

**Keywords:** Dental white spots, Dental enamel, Quaternary ammonium compounds, Biofilms, Composite resins, Dental caries

## Abstract

**Objectives:**

Considering the fact that resin infiltrants lack antibacterial activity, this study assessed the influence of the quaternary ammonium monomer dimethylaminohexadecyl methacrylate (DMAHDM) and amorphous calcium phosphate nanoparticles (NACP) on the physical and antibacterial properties of an experimental resin infiltrant (ERI).

**Methodology:**

The following groups were established: ERI (75/25 wt.% TEGDMA/BISEMA), ERI + 2.5% DMAHDM (2.5DM), ERI + 5% DMAHDM (5DM), ERI + 2% NACP (NACP), ERI + 2.5% DMAHDM + 2% NACP (2.5DM_NACP), ERI + 5% DMAHDM + 2% NACP (5DM_NACP), and Icon^®^ (IC), a commercial resin infiltrant. Degree of conversion (DC; n=4), sorption and solubility (SO/SOL; n=8), and contact angle (CA; n=10) tests were conducted. Biofilm biomass (BB; n=6) and bacterial metabolism (BM; n=8) were evaluated after *Streptococcus mutans* (UA159) cultivation for 48 h on material samples. Data were evaluated by one-way ANOVA and Tukey or Games-Howell *post hoc* tests (α=0.05).

**Results:**

IC exhibited the highest DC, with no difference from 2.5DM and 5DM. IC showed the lowest CA. IC had the lowest SO, followed by ERI, which had the lowest SOL, with no difference from IC. 5DM_NACP showed the lowest biofilm biomass, similar to 2.5DM and 5DM. Resin infiltrants containing DMAHDM showed reduced bacterial metabolism.

**Conclusions:**

DMAHDM, with or without NACP, demonstrated significant antibacterial activity, while NACP impaired DC. Both DMAHDM and NACP increased the contact angle, sorption, and solubility of the resin infiltrant, which may affect the material’s clinical performance.

## Introduction

Dental caries is a global health issue and one of the most prevalent oral diseases globally.^1^ It is a biofilm-sugar dependent disease that involves the interaction between acidogenic bacteria and dietary carbohydrate glycolysis, leading to the demineralization of dental hard tissues.^2^ Initial caries lesions present a whitish areas due to mineral loss from dental enamel, resulting in a porous lesion without cavitation.^2^

The ideal management of these initial lesions involves non-invasive treatments, such as reinforcing hygiene protocols and applying fluoride or remineralizing agents to arrest the lesion.^3^ However, in cases of non-compliance or interproximal lesions (where hygiene demands greater care) resin infiltrants are effective options for minimally invasive management, especially in combination with reinforcement of non-invasive protocols.^4,5^ Resin infiltrant is a low-viscosity material that fills the pores of initial caries lesions, sealing pathways of bacterial acid penetration and thus preventing lesion progression.^5^ Its effectiveness in controlling the progression of initial interproximal caries lesions was demonstrated in a randomized clinical trial with seven years of follow-up.^5^

Despite its advantages, it has been shown that the level of acid-producing bacteria in biofilms on resinous material surfaces is significantly higher than that on amalgam and glass ionomer materials,^6,7^ and the development of recurrent caries lesions at the material margins is the main cause associated with restorative procedure failures.^8^ Furthermore, this bacterial activity also degrades the restorative material.^9^

Methacrylate in resinous materials allows the binding of salivary proteins and bacterial colonizers.^6,7^ Therefore, there is a significant need to develop restorative materials that suppress this bacterial activity. Mechanisms that inhibit these bacterial processes can reduce the occurrence of recurrent caries lesions, and the development of materials with antibacterial and remineralizing properties has shown promising results.^10,11^

Quaternary ammonium monomers (QAMs) have high antibacterial efficacy and have been widely investigated in association with dental resinous materials.^12-15^ These monomers result from combining quaternary ammonium salts (QAS) with a methacrylate group. The structure of QAS is responsible for its antibacterial capacity, while the aliphatic vinyl group enables copolymerization with conventional dental monomers.^12-15^ Thus, QAMs are immobilized within the polymer structure through chemical bonding and will not be released or lost after curing, ensuring long-term antibacterial activity.^13,15^ The dimethylaminohexadecyl methacrylate (DMAHDM) monomer is a long-chain QAM (CL=16) that exhibits high efficacy against oral bacteria.^12,13,16^

The incorporation of bioactive particles in resinous materials has also shown promising results. The presence of nanoparticles of amorphous calcium phosphate (NACP) in these materials assists in creating a favorable environment for remineralization by releasing Ca and P ions, neutralizing acid, and increasing local pH.^15,17,18^ The combined incorporation of the therapeutic agents DMAHDM and NACP has a positive effect on the performance of restorative resinous materials, improving physical properties, exhibiting potent antibacterial and remineralizing activity, and enhancing the resistance of the materials to mechanical and acid challenges, thereby reducing the cariogenic impact of bacterial biofilms.^10,12,16,19^ A positive synergistic effect resulting from this association has been reported, as the ion release promoted by the NACP helps prevent demineralization and facilitate remineralization, further reducing the potential for secondary caries.^18^

Based on the positive results observed from the individual or combined use of these materials,^10-19^ it is relevant to investigate whether their inclusion in resin infiltrants could contribute to the development of a material with satisfactory properties, capable of establishing an environment unfavorable to the emergence of secondary caries and aligned with the principles of Minimally Invasive Dentistry.

Given the lack of equivalent studies in the literature, this study aimed to evaluate the effects of combining the antibacterial monomer DMAHDM with bioactive NACP in an experimental resin infiltrant. The following null hypotheses were tested: 1) The incorporation of DMAHDM and NACP agents, separately or combined, will not result in changes in the physical properties of resin infiltrants compared with those of other groups; 2) The incorporation of DMAHDM, by itself or in combination with NACP, will not result in antibacterial effects compared to other groups.

## Methodology

### Synthesis of Dimethylaminohexadecyl Methacrylate (DMAHDM)

DMAHDM was synthesized using a modified Menschutkin reaction method, as previously described.^20^ For synthesis, 10 mmol of 2-(dimethylamino)ethyl methacrylate (DMAEMA, Sigma-Aldrich, St. Louis, MO, USA), 10 mmol of 1-bromododecane (BDD, Sigma-Aldrich, St. Louis, MO, USA), and 3 g of ethanol were added to a flask, which was closed and stirred using a magnetic stir bar at 70°C for 24 h.

The material was placed under vacuum with constant rotation in a rotary evaporator (Rotavapor^®^ R-215, Büchi Labortechnik AG) to evaporate the solvent, removing unreacted components and impurities, yielding DMAHDM as a colorless and viscous liquid. Its structure was confirmed using Nuclear Magnetic Resonance Spectroscopy (NMR) and High-Resolution Mass Spectrometry (HRMS). NMR analysis was performed using ^1^H and ^13^C spectra, at 270 MHz resolution, at room temperature, with a sample rotation of 15 Hz, a tip angle of 45° for the observation pulse, and a recycle delay of 10 s, for 64 scans.^14^

### Formulation of experimental resin infiltrants

Experimental resin infiltrants (ERIs) were formulated using a monomeric mixture of 75% wt. TEGDMA and 25% wt. BisEMA, in addition to 0.5% wt. camphorquinone and 1% wt. ethyl 4-dimethylaminobenzoate (EDMAB). This monomeric composition was divided into pure ERI, groups containing 2.5% wt. (2.5DM) or 5% wt. (5DM) DMAHDM, a group containing 2% wt. NACP (NACP), and groups containing 2.5% wt. or 5% wt. DMAHDM + 2% wt. NACP (2.5DM_NACP and 5DM_NACP). NACP was sourced from Sigma Aldrich (Steinheim, Germany). The infiltrants were prepared under yellow light at 25°C. Additionally, a commercial resin infiltrant (Icon^®^, DMG - Germany) was tested (Table 1).

**Table 1:** Groups and their composition based on the therapeutic agents used by weight

Group	Composition (wt%)
ERI	98.5% TEGDMA + BisEMA blend; 0.5% CQ; 1% EDMAB
2.5DM	96% TEGDMA + BisEMA blend; 0.5% CQ; 1% EDMAB; 2.5% DMAHDM
5DM	93.5% TEGDMA + BisEMA blend; 0.5% CQ; 1% EDMAB; 5% DMAHDM
NACP	96.5% TEGDMA + BisEMA blend; 0.5% CQ; 1% EDMAB; 2% NACP
2.5DM_NACP	94% TEGDMA + BisEMA blend; 0.5% CQ; 1% EDMAB; 2.5% DMAHDM; 2% NACP
5DM_NACP	91.5% TEGDMA + BisEMA blend; 0.5% CQ; 1% EDMAB; 5% DMAHDM; 2% NACP
IC	Icon^®^: Triethylenglycoldimethacrylat-(TEGDMA) based resin matrix (about 78%). trimethylolpropantriacrylat (20%). campherchinon (<1%). (2-ethyl-hexyl)-p-dimethylaminobenzoat (<1%).2.6-di-tert-butyl-4-methylphenol (<1%). initiators*

*According to the manufacturer (DMG, Hamburg, Germany). The components were acquired from Sigma-Aldrich (Steinheim, Germany), except for BisEMA (Esstech, Essington, PA, USA). TEGDMA = triethylene glycol dimethacrylate, BisEMA = ethoxylated bisphenol A glycidyl dimethacrylate, CQ = camphorquinone, and EDMAB = ethyl 4-(dimethylamino)benzoate.

The experimental infiltrants were mixed at 120 rpm using a magnetic stirrer for 1 h. All infiltrants were stored in opaque containers, protected from light, and refrigerated.

### Sample size calculation

Sample size was estimated *a priori* using the G*Power software version 3.1.9.4, based on one-way ANOVA parameters and data extracted from a pilot study that evaluated water sorption and solubility variables. The input parameters were effect size (*f*=0.99), power (β=0.95), alpha error (*p*=0.05), and a dropout rate of 30%. The minimum required size was 7 sample units per group.

### Degree of conversion

Degree of conversion was analyzed via Fourier-Transform Infrared Spectroscopy (Vertex 70; Bruker Optik GmbH, Ettlingen, Germany) with Attenuated Total Reflectance (FTIR-ATR; MIRacle, Pike Technologies, Inc., Madison, WI, USA) by comparing the ratio of absorption bands at 1716 cm^-1^ and 1638 cm^-1^.^21^ An apparatus was adapted with masking tape on both sides of the crystal to ensure a standardized thickness and the unpolymerized material (n=4; 60 µl) was dispensed using a precision pipette (Microraman M25, Gilson Medical Electronics S.A., France) directly onto the ATR device’s crystal, coupled to the FTIR. A polyester strip was then placed over the material, followed by a glass slide. Then, the unpolymerized material was read. Subsequently, photoactivation was performed for 40 s (1000 mW/cm^2^ irradiance; Valo, Ultradent, South Jordan, UT, USA), and the polymerized material was read after 3 min. The degree of conversion was calculated from the polymerized-to-unpolymerized ratio according to the following formula:

### Water sorption and solubility

Water sorption (SO) and solubility (SOL) tests followed the ISO 4049/2019 specifications, except for the specimens’ dimensions.^22,23^ Using polyvinyl siloxane molds, disks (5 mm × 1 mm, n=8) of each material were fabricated. The infiltrants were deposited into the molds and light-cured using an LED light source for 60 s (1000 mW/cm^2^ irradiance; Valo, Ultradent, South Jordan, UT, USA), then placed in a desiccator containing silica gel, and stored in an oven at 37°C. The specimens were weighed every 24 h using an analytical balance (Shimadzu – AUW220D, Tokyo, Japan) until constant mass values (M1) were obtained, with a variation <0.002 g.

The volumes (mm^3^) of the specimens were obtained using their mean thickness and diameter measurements, with a digital caliper (Mitutoyo, Japan). After that, the specimens were stored for seven days in closed Eppendorf tubes containing 1.5 ml of distilled water at 37°C. The Eppendorf tubes were then removed from the oven and stored at room temperature for 30 min. The specimens were reweighed on the analytical balance, after being rinsed in running water and gently dried with absorbent paper to obtain M2 values. Once the M2 values were obtained, the specimens were stored again in the desiccator and weighed every 24 h until new constant mass values (M3) were found. The following formulas were used to calculate the values of SO and SOL:

### Contact angle

The mean contact angles (CA; n=10) for the materials were obtained by dispensing droplets onto glass slides, which were analyzed using a goniometer. The infiltrants were stored in syringes coated with insulating tape, and the glass slides were cleaned with absolute alcohol and dried in an oven at 37°C for 24 h. A new slide was used for each test. Droplets (~1 μL) were dispensed from a syringe positioned perpendicular to the slide, and images were captured with a camera attached to the goniometer (Ramé-hart-500F1, Succasunna, NJ, USA). The angles formed between the droplet and the surface were measured using specialized software for droplet shape analysis (DROPimage Advanced; Ramé-hart-500F1, Succasunna, NJ, USA). The average contact angles (degrees) on each side of the droplet were calculated for all groups.

### Bacterial biomass quantification assay

Disk-shaped specimens (n=6; 5 mm in diameter × 2 mm in thickness) were prepared from polyvinyl siloxane matrices.^11^ The infiltrants were deposited into the molds and light-cured with a LED light source for 60 s (1000 mW/cm^2^ irradiance; Valo, Ultradent, South Jordan, UT, USA). To standardize surface roughness, the specimens were polished using silicon carbide sandpapers of #600, #1200, and #2000 grit.

A strain of *Streptococcus mutans* (UA159) was used to promote biofilm formation on the specimens. The specimens were positioned in apparatuses and placed into wells of polystyrene microculture plates. Each well contained 300 μL of bacterial suspension at 10^8^ CFU/mL (adjusted to 0.1; 660 nm), 150 μL of 20% sucrose solution, and 2,550 μL of Brain Heart Infusion (BHI) agar medium. Biofilm was incubated for 48 h, with a change of medium after 24 h.^11^

After incubation, the specimens were washed with phosphate-buffered saline (PBS) and placed in a 24-well plate with 1 mL of 100% methanol for 15 min for fixation.^11^ The specimens were then washed again with PBS and transferred to another 24-well plate containing 1 mL of 0.1% crystal violet solution, where they rested for 5 min. The residual dye was removed by washing with PBS, and the specimens were transferred to another 24-well plate, where 2 mL of 95% ethanol solution was added to each well. The plate was horizontally shaken at 80 rpm at room temperature for 45 min.^11^

The ethanol solution (100 μL) from each well was diluted with 95% ethanol solution to 200 μL and transferred to a 96-well plate. The solution absorbance was measured in a microplate reader (ASYS-UVM 340, Biochrom Ltd, Cambridge, England) at OD 595 nm.^11^

### Bacterial Metabolism assay

Disk-shaped specimens (n=8; 5 mm in diameter × 2 mm in thickness) were prepared and polished as previously described for the Bacterial Biomass Quantification Assay, and was the case for the *S. mutans* biofilm incubation. After 48 h of incubation, the specimens were washed with PBS, transferred to a 24-well plate containing 2 mL of MTT (3-[4,5-dimethylthiazol-2-yl]-2,5-diphenyltetrazolium bromide) at 0.5 mg/mL per well, and incubated at 37 °C in 5% CO^2^for 1 h. Then, the specimens were transferred to another 24-well plate with 2 mL of dimethyl sulfoxide (DMSO) in each well and incubated under horizontal agitation (80 rpm) at room temperature for 20 min to dissolve the formazan crystals. After that, 200 μL of the solution from each well were transferred to a 96-well plate, and the optical density of 540 nm was determined using a microplate reader (ASYS-UVM 340, Biochrom Ltd, England).^11,24^

### Statistics analysis

Normality and homogeneity of variance were tested using Shapiro–Wilk and Levene’s tests, respectively. The data from the degree of conversion, contact angle, and water sorption tests were analyzed by one-way ANOVA followed by Tukey’s post-hoc test, while the data from the water solubility, bacterial biomass quantification, and bacterial metabolism tests were analyzed using Welch’s ANOVA with the Games-Howell post-hoc test. A significance level of 5% (p<0.05) was adopted for all analyses (IBM SPSS Statistics for Windows, version 20.0. Armonk, NY: IBM Corp).

## Results

The mean and standard deviation values for degree of conversion, water sorption and solubility, and contact angle across the groups are described in Table 2.

**Table 2:** Mean and standard deviation (SD) of the resin infiltrants for the variables degree of conversion, water sorption, water solubility, and contact angle

Variable	Resin infiltrant							
	ERI	2.5DM	5DM	NACP	2.5DM_NACP	5DM_NACP	IC	p value
Degree of conversion (%)	40.0 (4.07) BC	47.72 (8.98) ABC	53.22 (14.84) AB	31.64 (2.85) C	34.65 (2.85) BC	39.84 (2.23) BC	60.4 (10.99) A	0.001
Water sorption (µg/mm^3^)	77.3 (4.44) D	105.11 (5.6) C	109.51 (3.92) BC	104.46 (8.06) C	125.24 (11.79) A	118.31 (6.85) AB	47.14 (2.73) E	<0.001
Water solubility (µg/mm^3^)	3.48 (1.76) D	37.9 (9.7) AC	22.38 (3.19) B	52.39 (8.06) A	43.82 (12.73) A	25.83 (5.12) BC	9.69 (5.53) D	<0.001
Contact angle (OE^0^)	30 (2.65) D	30.23 (0.91) CD	37.26 (3.41) A	31.64 (4.00) BCD	34.45 (2.99) AB	33.96 (2.42) AC	25.66 (2.55) E	<0.001

### Degree of conversion

The IC group had the highest mean value, with no statistically significant difference only from the 2.5DM and 5DM groups. Among the experimental groups, 5DM had the highest mean, differing only from the NACP group, which had the lowest mean. The NACP group differed only from the 5DM and IC groups.

### Water sorption and solubility

For water sorption, the IC group had the lowest mean values, differing significantly from all other groups. The ERI (pure experimental) group had the second lowest mean, also differing from all others. The 2.5DM_NACP group had the highest mean, differing from all other groups except for 5DM_NACP, which had the second highest mean.

For water solubility, the ERI group had the lowest mean, differing statistically from all other groups except for IC, which had the second lowest mean and differed from all others except for ERI. NACP had the highest mean among the groups, with no statistically significant difference only from the 2.5DM and 2.5DM_NACP groups.

### Contact angle

The IC group had the lowest mean, differing from all others. The 5DM group had the highest mean values, with no statistically significant difference from the 2.5DM_NACP and 5DM_NACP groups. The 2.5DM and NACP groups did not differ from the pure experimental group (ERI).

### Biofilm biomass accumulation

**Figure 1:**
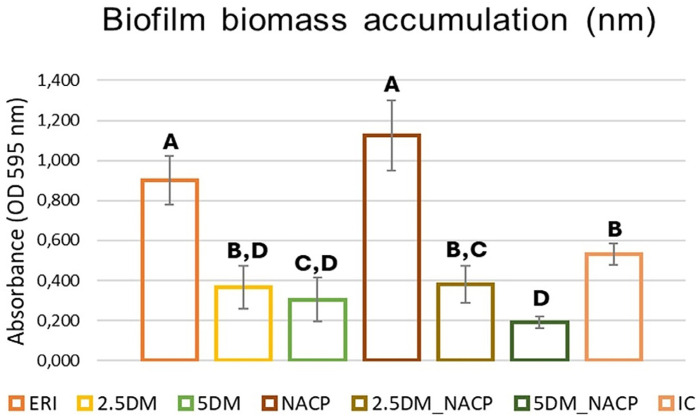
Mean and standard deviation results of biofilm biomass accumulation (nm) per group. Different letters indicate statistical differences among the groups (p<0.05).

The mean and SD values of biofilm biomass accumulation are described in Figure 1. The NACP group had the highest mean values, differing from all others except for ERI. The ERI group had the second highest mean values, also differing from all others except for NACP. The 5DM_NACP group had the lowest mean values, with no statistically significant difference from only the 2.5DM and 5DM groups.

### Bacterial metabolism

**Figure 2:**
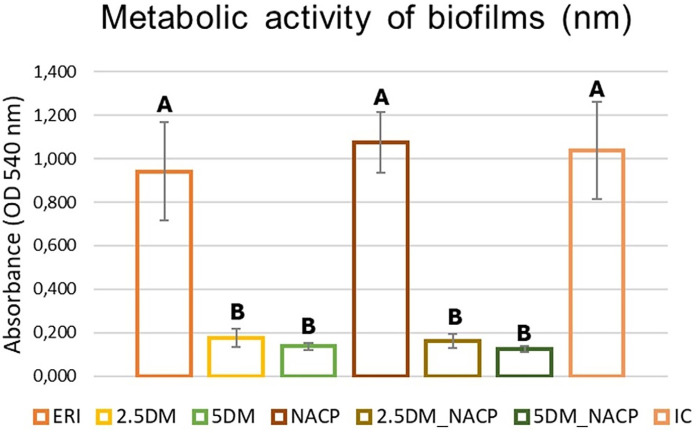
Mean and standard deviation results of bacterial biofilm metabolism (nm) per group. Different letters indicate statistical differences among the groups (p<0.05).

Figure 2 shows the mean and SD values of bacterial metabolism. The ERI, NACP, and IC groups, which do not contain the DMAHDM monomer, had the highest mean values, showing no statistically significant differences from each other but differing from all other groups. The 2.5DM, 5DM, 2.5DM_NACP, and 5DM_NACP groups did not differ from each other*.*

## Discussion

This study investigated novel compositions of resin infiltrants containing DMAHDM and NACP to reduce bacterial activity on the material surface. The null hypotheses were rejected, as the addition of the agents influenced the physical properties and resulted in antibacterial effects when compared to the control groups.

The addition of DMAHDM by itself did not impair the degree of conversion of the materials, as no statistically significant difference was found compared to the commercial resin infiltrant. In contrast, the addition of NACP resulted in the lowest values. Previous studies have reported that the incorporation of DMAHDM did not impair the degree of conversion of resin materials, which may be due to its ability to copolymerize with the resin matrix.^12,15,16,19^ DMAHDM is a monomethacrylate with reactive groups on one end of the molecule, allowing its incorporation into the resin with less negative impact on the mechanical properties.^25^

However, it has been reported that the addition of short-chain QAMs tends to result in higher degrees of conversion than long-chain ones, such as DMAHDM, potentially due to the greater mobility of monomers with shorter alkyl chains.^26,27^ A previous study using thermogravimetric analysis observed that the addition of DMAHDM might affect the glass transition temperature due to the monomer’s long chain, influencing the polymerization characteristics and chain formation.^27^ In our study, the changes observed in the 2.5DM and 5DM groups, which contained only DMAHDM, were insufficient to impair the DC. However, the addition of NACP resulted in a significant impairment of DC, which could not be counterbalanced by the association with DMAHDM in the 2.5DM_NACP and 5DM_NACP groups, possibly due to the interaction between the organic and inorganic phases.^27^

Previous studies that incorporated NACP into resin infiltrants^28^ or adhesives^12^ found that the addition did not impair, or even improve, the DC of the materials. Such studies used NACP concentrations of 10-20% wt., considerably higher than the 2% wt. concentration used in this study. According to the literature, a mismatch between the refractive index (RI) of the particles and the resin matrix can hinder light transmittance through the material, which impairs polymerization.^29^ Furthermore, particle agglomeration may have contributed to increased light scattering.^29^

For water sorption and solubility, the groups without added components exhibited the lowest rates, while those containing the combination of DMAHDM and NACP showed the highest. Sorption and solubility are related to various chemical and physical processes that can result in impairments, such as volumetric changes and physical and chemical modifications in the structure and function of polymers.^27,30^

Water sorption appears to be dictated by the polarity of the composite. Considering that QAS molecules are both hydrophilic and nonpolar,^27^ it can be expected that the higher the QAS content in the material composition, the more hydrophilic the composite will be.^27^ Another factor is the heterogeneity of the resulting polymer network due to the presence of added therapeutic agents, which, coupled with hydrophilicity, may have contributed to the increase in water sorption.^23,27^ Increased water sorption and solubility after the incorporation of DMAHDM or bioactive particles into the resin material have been observed in previous studies.^23,27^

In addition, the monomer BISEMA 30, a longer version of BISEMA used in the monomeric base of the experimental resin infiltrants, exhibits high sorption. This may be due to the increased polarity of the hydrophilic site of the molecule, resulting from the presence of long ethylene oxide chains.^31^ The BISEMA 30 monomer has a higher molecular weight than the conventional one because of the longer ethylene oxide spacer, which provides greater flexibility, making it the monomer of choice for the composition of the experimental infiltrants.^31^ In a previous study, the use of BISEMA 30 associated with TEGDMA showed a higher degree of conversion compared with the association of TEGDMA with regular BISEMA.^31^

However, high sorption and solubility were observed for the BISEMA 30-TEGDMA mixture despite the high degree of conversion of the material.^31^ It was reported that the presence of a larger and more flexible spacer, along with the absence of the secondary hydrogen functional group that would promote strong molecular interactions, may be responsible for the formation of a highly flexible polymer network with a high free volume due to impaired molecular packing.^31^ This impaired polymer network would then facilitate passive solvent diffusion through the material, resulting in network expansion and high mobility of the unreacted monomers, ultimately leading to their leaching and high solubility.^31^

High sorption rates are directly related to increased solubility because the polymer network, swollen by water absorption, enables the release of unreacted monomers and soluble NACP from the material.^23,27^ A previous study observed that increasing the amount of QAS, especially for long-chain ones like DMAHDM, also increases the hydroscopic expansion of the resulting material, which could be related to the lower cross-linking density of the polymer network formed as a result of the concentration and chain size.^27^

Other studies consistent with these results have reported that resin materials formulated with QAS are more susceptible to chemical degradation.^27^ An earlier study found that composite resins containing DMADDM or DMAHDM at 5% or 10% showed a reduction in Knoop microhardness, which is an indirect indicator of cross-linking density, after 14 days of storage in ethanol.^27^ While the experimental and commercial control groups are mainly composed of methacrylates (which can form dense, cross-linked networks), composites containing QAS showed greater susceptibility to degradation. This may be due to the more linear networks derived from methacrylates, allowing solvent penetration. The mechanisms underlying this behavior may be solvent penetration and chain breakage of the polymer matrix, as the attractive forces between the polymer networks are overcome by the attractive forces between the solvent molecules and the chains.^27,30^

The commercial group exhibited the lowest mean contact angle, which differed from all others. The 5DM group had the highest mean values, not significantly different from the 2.5DM_NACP and 5DM_NACP groups. The 2.5DM and NACP groups did not differ from the pure experimental group (ERI). As the resin infiltrant does not contain any fillers in its composition, the addition of components, even in low concentrations, can increase its viscosity; this, in turn, leads to an increase in the surface tension coefficient and a reduction in wettability.^23^ Previous studies that added particles to the same resin matrix we investigated also observed an increase in the contact angle. However, this increase did not impair the penetration of the infiltrants into initial carious lesions in human enamel *in vitro* and, therefore, may not be a drawback.^23^ NACP was incorporated into the material due to its ability to release high levels of Ca and P ions at acidic pH when these ions would be most needed to combat caries. The ion release from the materials will be further tested.

The groups containing DMAHDM exhibited potent antibacterial activity against *Streptococcus mutans*. In this study, *S. mutans* was selected for antimicrobial assays because of its significance as a bacterium associated with dental caries. This is attributed to its high acid production capacity, acid tolerance, and extensive synthesis of both intra- and extra-cellular polysaccharides, which enable it to form an aggressive biofilm and acidify the local plaque environment, ultimately leading to demineralization.^32^

Biofilm biomass accumulation was evaluated using a method based on staining with crystal violet, a basic dye that binds to negatively charged surface molecules and polysaccharides in the extracellular matrix, serving as an indicator of bacterial growth.^33^ The biofilm was treated and incubated with crystal violet solution, which was absorbed by live bacterial cells and adhered to the surface of the cell membrane.^24,33^ The intensity of the color associated with the biofilm is measured using spectrophotometry, and higher absorbance means greater biomass, suggesting lower antibacterial activity. Conversely, a decrease in color intensity is associated with bactericidal activity.^33^

To assess the metabolic activity of bacteria, a colorimetric assay using MTT (3-[4,5-dimethylthiazol-2-yl]-2,5-diphenyltetrazolium bromide) was performed. This assay is based on the ability of live cells to reduce MTT to formazan, a product insoluble in water that imparts an intense purple.^11,24^ Quantifying the absorbance obtained in the MTT assay allows for the estimation of cell viability, as the amount of formazan formed is directly related to the number of living cells. Higher absorbance values indicate a higher formazan concentration, reflecting greater metabolic activity in the biofilm.^11,24^

Previous studies demonstrated that the addition of a QAM to resin infiltrants promotes antibacterial activity against a multi-species biofilm containing *S. mutans, S. sanguinis,* and *S. gordonii*.^11^ Yu, et al.^11^ (2020) evaluated the incorporation of the QAM dimethylaminododecyl methacrylate (DMADDM) into Icon^®^, a commercial resin infiltrant, at concentrations of 0%, 2.5%, 5%, and 10%. The researchers used these materials to treat initial caries lesions induced in bovine enamel and conducted various tests, observing that biofilm accumulation significantly decreased as the mass fraction of DMADDM increased. The incorporation significantly reduced the ratio of live-to-dead bacteria, biomass production, lactic acid, and extracellular polysaccharides of the bacterial biofilm when compared to the control group without QAM, without influencing the color properties, surface morphology, surface roughness, and biocompatibility, even after 1 month of microbial aging.^11^

The aforementioned study also evaluated the anti-demineralization effect of the infiltrants through transverse microradiography (TMR) after 48 h of biofilm demineralization treatment. Compared to the control group without DMADDM, the depth and amount of demineralization were significantly reduced in the groups containing the monomer ^11^. While DMADDM has an alkyl chain length (CL) of 12, the more recently developed DMAHDM, employed in this research, has CL=16. Longer chains increase the antibacterial effectiveness of the monomer.^34^

Bhadila, et al.^15^ (2021) corroborates our results by observing that the incorporation of 3% DMAHDM and 20% NACP in a composite resin based on triethylene glycol divinylbenzyl ether (TEG-DVBE), urethane dimethacrylate (UDMA), and 43-45% glass filler significantly reduced the acid-producing ability of *S. mutans*, thus raising the pH from a cariogenic 4.7 to a safe 7.1. The composite containing DMAHDM showed much lower biofilm biomass production (including live and dead cells and extracellular matrix) than the controls, and did not present a decline in mechanical properties.^15^ As the new nanocomposite has ion release and antibacterial benefits, it is a promising alternative to help reduce secondary caries at margins and increase the longevity of restorations.

Previous studies have reported that increasing the mass fraction of QAM enhances its antibacterial activity.^16,35^ Studies conducted with fractions of 0.75-5% DMAHDM have reported efficacious bacterial suppression without impairing the properties of the resulting resin materials.^16,35^ However, Filemban, et al.^16^ (2022) reported impairments in flexural strength and modulus of elasticity in additions >5%. These authors also used a polymer base matrix composed of UDMA, TEG-DVBE, and 45-65% glass filler and reported that increasing the mass fraction of DMAHDM above 5% led to a viscous resin and a less cohesive resin composite paste.

The reported increase in biofilm suppression activity associated with the rise in the mass fraction of DMAHDM can be attributed to an increase in charge density.^16,19^ Increasing the positive charge of DMAHDM enables interaction with more negatively charged bacterial membranes, which may lead to disruption of membrane functions and imbalance of the K^+^, Na^+^, Ca^2^, and Mg^2^ essential ions, followed by bacterial lysis.^16,19^

Resin materials are subject to degradation, which increases as the filler rate decreases, as reported by previous studies^36^_._ This is particularly concerning for resin infiltrants, whose composition does not include fillers. The TEGDMA monomer contains two hydrolysable ester groups, and these ester linkage groups can separate by either hydrolysis or acid-, base-, or enzyme-induced saponification in the intraoral environment. This may especially occur at the interface between the material and the dental substrate^37^. Esterase, an enzyme found in human saliva as well as secreted by cariogenic bacteria such as *S. mutans*, can break down these ester groups^9^. Esterase is a hydrolase enzyme that cleaves esters into an alcohol and a carboxyl portion through a chemical reaction with water, known as hydrolysis.^9^

The breakdown of ester groups in TEGDMA produces methacrylic acid (MA) and two other byproducts:^38^ 2-(2-(2-(2-hydroxyethoxy)ethoxy)ethoxy)ethyl methacrylate (TEGMA), produced when the first ester group is cleaved, and TEG, generated when both ester groups are hydrolyzed.^38^ This hydrolysis causes polymer mass loss and resin softening, and the accumulation of this degradation due to constant exposure to the thermal, mechanical, and biochemical challenges of the oral cavity may lead to material integrity loss, resulting in staining and recurrent caries.^37,38^

It has also been suggested that degradation byproducts of dental monomers, such as TEGDMA and BISGMA, can alter the metabolism and promote the proliferation *of S. mutans* and biofilm formation.^39^ The acid production by bacteria in this biofilm can alter the material surface, increasing surface roughness and porosity, which may in turn increase bacterial adhesion and accumulation.^40^ Therefore, the development of dental materials with antimicrobial potential may assist in reducing the occurrence of these lesions.

The use of resin infiltrants with antibacterial activity may be particularly beneficial for non-compliant patients and groups at higher risk for caries, such as children, older adults, and those with special needs. Developing multifunctional composites is challenging due to the potential compromise of the materials’ properties; as such, different formulations must be investigated to obtain materials that exhibit favorable characteristics to clinical longevity. One limitation of this study is the *in vitro* evaluation of the materials, which does not fully simulate the complexity of oral conditions. Additionally, the ionic release capacity due to the presence of NACP and its potential benefit in creating an anticariogenic environment warrants further investigation.

Within the limitations of this study, it was observed that adding the investigated agents to the resin infiltrant produced a material with significant antibacterial efficacy. However, the high sorption and solubility of the resulting material may compromise its clinical longevity. Future studies should investigate whether lower concentrations of the antibacterial monomer would result in significant antibacterial efficacy with less damage to the polymer network, or whether different monomers in the composition of the resin matrix in the resin infiltrant could result in better properties.

## Conclusions

The incorporation of the DMAHDM monomer at 2.5% or 5%, with or without 2% NACP, into the experimental resin infiltrant demonstrated potent antibacterial activity. However, this addition compromised the sorption and solubility of the resulting material, as well as the degree of conversion of the infiltrants. Changes in physical properties may affect the longevity and clinical performance of the materials.
